# Diethyl 2-(triphenyl­meth­yl)malonate

**DOI:** 10.1107/S1600536808018163

**Published:** 2008-06-21

**Authors:** Xian-You Yuan, Min Zhang, Seik Weng Ng

**Affiliations:** a Department of Biology and Chemistry, Hunan University of Science and Engineering, Yongzhou 425100, People’s Republic of China; b Department of Chemistry, University of Malaya, 50603 Kuala Lumpur, Malaysia

## Abstract

In the title compound, C_26_H_26_O_4_, steric crowding of the Ph_3_C– group with the –CH(CO_2_Et)_2_ unit leads to a long C—C bond [1.585 (2) Å]. One of the two ethyl groups is disordered over two sites in a 60:40 ratio.

## Related literature

For the synthesis by direct reaction of trityl chloride with diethyl malonate, see: Patai *et al.* (1962 ▶). For the medicinal use of the compound, see: Brugnara *et al.* (2000 ▶); Lencer *et al.* (2002 ▶). For a related crystal structure, 3-(triphenyl­meth­yl)-2,4-penta­dione, see: Sykora *et al.* (2007 ▶).
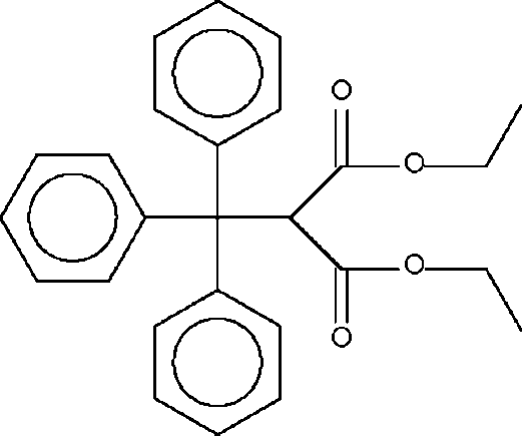


## Experimental

### 

#### Crystal data

C_26_H_26_O_4_
*M* *_r_* = 402.47
Monoclinic, 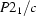

*a* = 8.5871 (5) Å
*b* = 19.884 (1) Å
*c* = 12.7591 (8) Å
β = 97.302 (1)°
*V* = 2160.9 (2) Å^3^
*Z* = 4
Mo *K*α radiation
μ = 0.08 mm^−1^
*T* = 295 (2) K
0.48 × 0.45 × 0.40 mm

#### Data collection

Bruker SMART 1000 diffractometer
Absorption correction: none
12570 measured reflections
4693 independent reflections
2761 reflections with *I* > 2σ(*I*)
*R*_int_ = 0.045

#### Refinement

*R*[*F* ^2^ > 2σ(*F* ^2^)] = 0.051
*wR*(*F* ^2^) = 0.150
*S* = 1.00
4693 reflections
290 parameters
28 restraints
H-atom parameters constrained
Δρ_max_ = 0.26 e Å^−3^
Δρ_min_ = −0.22 e Å^−3^

### 

Data collection: *SMART* (Bruker, 1997 ▶); cell refinement: *SAINT* (Bruker, 2003 ▶); data reduction: *SAINT*; program(s) used to solve structure: *SHELXS97* (Sheldrick, 2008 ▶); program(s) used to refine structure: *SHELXL97* (Sheldrick, 2008 ▶); molecular graphics: *X-SEED* (Barbour, 2001 ▶); software used to prepare material for publication: *publCIF* (Westrip, 2008 ▶).

## Supplementary Material

Crystal structure: contains datablock(s) global, I. DOI: 10.1107/S1600536808018163/bt2717sup1.cif

Structure factors: contains datablock(s) I. DOI: 10.1107/S1600536808018163/bt2717Isup2.hkl

Additional supplementary materials:  crystallographic information; 3D view; checkCIF report

## Figures and Tables

**Fig. 1.**
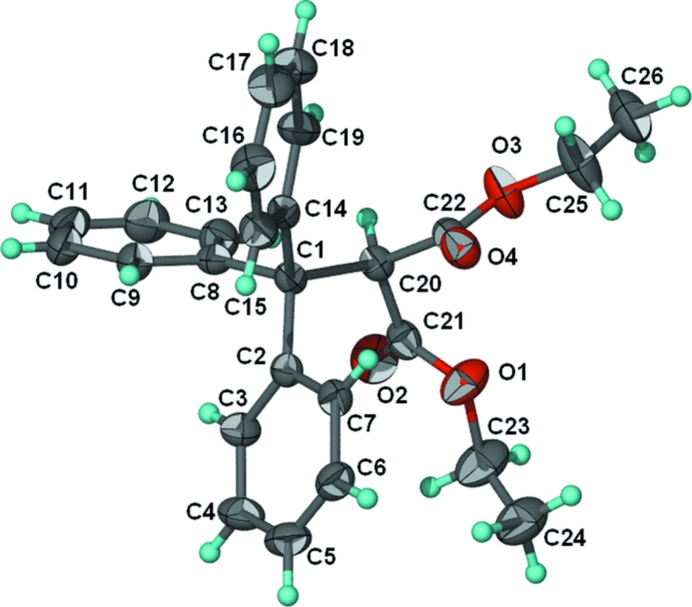
Thermal ellipsoid plot (Barbour, 2001) plot of at the 50% probability level. Hydrogen atoms are drawn as spheres of arbitrary radius. The minor disorder component of the ethyl group is not shown.

## supplementary crystallographic information

### Comment

The title compound can be synthesized by triturating triphenylmethyl chloride with diethyl malonate, which is an active methylene compound (Patai *et al.*, 1962). The compound belongs to a class of triarylmethane derivatives that have been patented for the treatment of diarrhoea (Lencer *et al.*, 2002) as well as for sickle-cell anemia (Brugnara *et al.*, 2000). The compound (Scheme I, Fig. 1) features crowding of the Ph_3_C– portion with the –CH(CO_2_Et)_2_ portion, which leads to a long carbon-carbon bond [1.585 (2) Å]. The distance is similar to that [1.587 (3) Å] found in 3-(triphenylmethyl)-2,4-pentadione (Sykora *et al.*, 2007).

### Experimental

To a solution of diethyl malonate (1.15 g, 7.18 mmol) in anhydrous ethanol (6 ml) was added powdered magnesium (0.18 g, 7.4 mmol) along with several drops of carbon tetrachloride and a granule of iodine. After the reaction has abated, the mixture was heated until the magnesium had dissolved completely. The solvent was allowed to evaporate. The solid material was ground with benzene (4 ml). The solvent was again evaporated. The residue was redissolved in benzene (4 ml), and to the solution was added triphenylmethyl chloride (2 g, 7.18 mmol) in benzene (4 ml). The mixture was stirred for five hours until it turned pale green. 10% Hydrochloric acid was added to dissolve the precipitate of magnesium hydroxide. The solution was extracted twice with benzene (4 ml). The combined organic portions were washed twice with water (5 ml) before being dried over an anhydrous salt. The solvent benzene was evaporated and the product recrystallized from acetone to afford colorless crystals (yield 75%); m.p. 411–412 K.

### Refinement

One of the two ethyl groups is disordered over two sites in a 0.60 (1):0.40 (1) ratio. The O–C distances were restrained to 1.45±0.01 Å and the C–C distances to 1.50 + 0.01 Å. The anisotropic displacement parameters of the disordered atoms were restrained an isotropic behaviour.

Carbon-bound H-atoms were placed in calculated positions (C—H 0.93 to 0.97 Å) and were included in the refinement in the riding model approximation, with *U*(H) set to 1.2 to 1.5*U*_eq_(C).

### Crystal data

**Table:**

C_26_H_26_O_4_	*F*(000) = 856
*M**_r_* = 402.47	*D*_x_ = 1.237 Mg m^−^^3^
Monoclinic, *P*2_1_/*c*	Mo *K*α radiation, λ = 0.71073 Å
Hall symbol: -P 2ybc	Cell parameters from 3695 reflections
*a* = 8.5871 (5) Å	θ = 2.6–27.1°
*b* = 19.884 (1) Å	µ = 0.08 mm^−^^1^
*c* = 12.7591 (8) Å	*T* = 295 K
β = 97.302 (1)°	Block, colorless
*V* = 2160.9 (2) Å^3^	0.48 × 0.45 × 0.40 mm
*Z* = 4	

### Data collection

**Table:**

Bruker SMART 1000 diffractometer	2761 reflections with *I* > 2σ(*I*)
Radiation source: medium-focus sealed tube	*R*_int_ = 0.045
graphite	θ_max_ = 27.1°, θ_min_ = 1.9°
φ and ω scans	*h* = −8→11
12570 measured reflections	*k* = −25→25
4693 independent reflections	*l* = −16→15

### Refinement

**Table:**

Refinement on *F*^2^	Primary atom site location: structure-invariant direct methods
Least-squares matrix: full	Secondary atom site location: difference Fourier map
*R*[*F*^2^ > 2σ(*F*^2^)] = 0.051	Hydrogen site location: inferred from neighbouring sites
*wR*(*F*^2^) = 0.151	H-atom parameters constrained
*S* = 1.00	*w* = 1/[σ^2^(*F*_o_^2^) + (0.0735*P*)^2^ + 0.2019*P*] where *P* = (*F*_o_^2^ + 2*F*_c_^2^)/3
4693 reflections	(Δ/σ)_max_ = 0.001
290 parameters	Δρ_max_ = 0.26 e Å^−^^3^
28 restraints	Δρ_min_ = −0.22 e Å^−^^3^

### Fractional atomic coordinates and isotropic or equivalent isotropic displacement parameters (Å^2^)

**Table:**

	*x*	*y*	*z*	*U*_iso_*/*U*_eq_	Occ. (<1)
O1	0.53297 (19)	0.54974 (7)	0.74906 (12)	0.0649 (4)	
O2	0.64769 (19)	0.50922 (8)	0.61546 (13)	0.0669 (5)	
O4	0.24156 (16)	0.46954 (7)	0.79704 (10)	0.0502 (4)	
O3	0.19910 (17)	0.51697 (8)	0.63787 (11)	0.0613 (4)	
C1	0.4903 (2)	0.38239 (8)	0.71672 (13)	0.0358 (4)	
C2	0.6123 (2)	0.39784 (8)	0.81297 (13)	0.0359 (4)	
C3	0.7724 (2)	0.39498 (10)	0.80592 (15)	0.0449 (5)	
H3	0.8065	0.3808	0.7432	0.054*	
C4	0.8819 (2)	0.41277 (11)	0.89050 (17)	0.0557 (5)	
H4	0.9885	0.4104	0.8842	0.067*	
C5	0.8340 (3)	0.43394 (10)	0.98370 (17)	0.0567 (6)	
H5	0.9076	0.4454	1.0408	0.068*	
C6	0.6760 (3)	0.43800 (10)	0.99189 (15)	0.0495 (5)	
H6	0.6428	0.4524	1.0547	0.059*	
C7	0.5668 (2)	0.42082 (9)	0.90763 (14)	0.0425 (4)	
H7	0.4605	0.4246	0.9141	0.051*	
C8	0.5680 (2)	0.33995 (9)	0.63526 (14)	0.0395 (4)	
C9	0.6181 (2)	0.27544 (9)	0.66570 (16)	0.0495 (5)	
H9	0.6047	0.2602	0.7329	0.059*	
C10	0.6866 (3)	0.23381 (11)	0.5992 (2)	0.0590 (6)	
H10	0.7203	0.1912	0.6220	0.071*	
C11	0.7059 (3)	0.25471 (12)	0.49889 (19)	0.0624 (6)	
H11	0.7513	0.2264	0.4534	0.075*	
C12	0.6575 (3)	0.31736 (13)	0.46735 (18)	0.0620 (6)	
H12	0.6697	0.3318	0.3995	0.074*	
C13	0.5898 (2)	0.36049 (11)	0.53507 (15)	0.0520 (5)	
H13	0.5591	0.4035	0.5123	0.062*	
C14	0.3499 (2)	0.33914 (9)	0.74171 (14)	0.0388 (4)	
C15	0.3452 (2)	0.30478 (9)	0.83520 (16)	0.0480 (5)	
H15	0.4302	0.3075	0.8881	0.058*	
C16	0.2164 (3)	0.26641 (11)	0.8516 (2)	0.0628 (6)	
H16	0.2153	0.2439	0.9154	0.075*	
C17	0.0903 (3)	0.26130 (12)	0.7744 (2)	0.0677 (7)	
H17	0.0027	0.2363	0.7863	0.081*	
C18	0.0944 (3)	0.29331 (11)	0.6800 (2)	0.0657 (6)	
H18	0.0101	0.2895	0.6269	0.079*	
C19	0.2231 (2)	0.33111 (10)	0.66349 (17)	0.0522 (5)	
H19	0.2253	0.3518	0.5983	0.063*	
C20	0.4235 (2)	0.45117 (9)	0.66689 (14)	0.0381 (4)	
H20	0.3912	0.4426	0.5916	0.046*	
C21	0.5482 (2)	0.50592 (9)	0.67315 (15)	0.0436 (5)	
C22	0.2799 (2)	0.47852 (9)	0.71124 (15)	0.0401 (4)	
C23	0.6698 (11)	0.5959 (4)	0.7676 (7)	0.072 (2)	0.60 (1)
H23A	0.6579	0.6324	0.7169	0.087*	0.60 (1)
H23B	0.7657	0.5718	0.7592	0.087*	0.60 (1)
C23'	0.6178 (13)	0.6129 (4)	0.7620 (10)	0.068 (3)	0.40 (1)
H23C	0.5493	0.6492	0.7782	0.082*	0.40 (1)
H23D	0.6640	0.6243	0.6988	0.082*	0.40 (1)
C24	0.6779 (11)	0.6230 (4)	0.8771 (4)	0.079 (2)	0.60 (1)
H24A	0.7610	0.6554	0.8888	0.118*	0.60 (1)
H24B	0.6979	0.5869	0.9269	0.118*	0.60 (1)
H24C	0.5799	0.6441	0.8861	0.118*	0.60 (1)
C24'	0.7417 (13)	0.5995 (6)	0.8527 (9)	0.079 (3)	0.40 (1)
H24D	0.8071	0.6386	0.8656	0.118*	0.40 (1)
H24E	0.8045	0.5620	0.8362	0.118*	0.40 (1)
H24F	0.6932	0.5895	0.9146	0.118*	0.40 (1)
C25	0.0595 (3)	0.54963 (15)	0.6682 (2)	0.0814 (8)	
H25A	−0.0127	0.5161	0.6889	0.098*	
H25B	0.0885	0.5793	0.7278	0.098*	
C26	−0.0156 (3)	0.58803 (13)	0.5784 (2)	0.0773 (8)	
H26A	−0.1078	0.6097	0.5975	0.116*	
H26B	−0.0446	0.5583	0.5199	0.116*	
H26C	0.0562	0.6214	0.5587	0.116*	

### Atomic displacement parameters (Å^2^)

**Table:**

	*U*^11^	*U*^22^	*U*^33^	*U*^12^	*U*^13^	*U*^23^
O1	0.0754 (11)	0.0514 (8)	0.0723 (10)	−0.0232 (8)	0.0262 (8)	−0.0127 (8)
O2	0.0595 (10)	0.0647 (10)	0.0821 (11)	−0.0104 (8)	0.0309 (9)	0.0031 (8)
O4	0.0437 (8)	0.0608 (9)	0.0471 (8)	0.0084 (6)	0.0096 (6)	0.0015 (6)
O3	0.0503 (9)	0.0776 (10)	0.0582 (9)	0.0294 (8)	0.0153 (7)	0.0177 (8)
C1	0.0308 (9)	0.0362 (9)	0.0399 (9)	0.0017 (7)	0.0028 (8)	0.0010 (7)
C2	0.0347 (10)	0.0307 (9)	0.0415 (10)	0.0007 (7)	0.0021 (8)	0.0036 (7)
C3	0.0365 (11)	0.0484 (11)	0.0495 (11)	−0.0053 (8)	0.0042 (9)	−0.0005 (9)
C4	0.0347 (11)	0.0638 (14)	0.0659 (14)	−0.0108 (10)	−0.0040 (10)	0.0004 (11)
C5	0.0565 (14)	0.0555 (13)	0.0535 (13)	−0.0110 (10)	−0.0113 (11)	−0.0013 (10)
C6	0.0590 (14)	0.0432 (11)	0.0448 (11)	−0.0002 (9)	0.0005 (10)	−0.0040 (9)
C7	0.0415 (11)	0.0397 (10)	0.0455 (10)	0.0051 (8)	0.0024 (9)	0.0015 (8)
C8	0.0308 (10)	0.0426 (10)	0.0439 (10)	−0.0025 (8)	−0.0001 (8)	−0.0066 (8)
C9	0.0451 (12)	0.0433 (11)	0.0595 (12)	0.0028 (9)	0.0039 (10)	−0.0058 (9)
C10	0.0479 (13)	0.0451 (12)	0.0829 (16)	0.0016 (10)	0.0045 (12)	−0.0185 (11)
C11	0.0435 (13)	0.0681 (16)	0.0755 (16)	−0.0016 (11)	0.0067 (11)	−0.0355 (13)
C12	0.0566 (14)	0.0766 (17)	0.0544 (13)	0.0005 (12)	0.0132 (11)	−0.0159 (12)
C13	0.0504 (13)	0.0555 (12)	0.0507 (12)	0.0050 (10)	0.0092 (10)	−0.0037 (10)
C14	0.0316 (10)	0.0362 (9)	0.0485 (11)	0.0021 (8)	0.0046 (8)	−0.0013 (8)
C15	0.0428 (11)	0.0437 (11)	0.0571 (12)	−0.0020 (9)	0.0044 (9)	0.0063 (9)
C16	0.0600 (15)	0.0523 (13)	0.0791 (15)	−0.0059 (11)	0.0201 (13)	0.0121 (11)
C17	0.0432 (13)	0.0574 (14)	0.1040 (19)	−0.0133 (10)	0.0153 (13)	0.0043 (13)
C18	0.0425 (13)	0.0626 (14)	0.0887 (17)	−0.0090 (10)	−0.0048 (12)	−0.0025 (13)
C19	0.0412 (12)	0.0524 (12)	0.0604 (12)	−0.0061 (9)	−0.0035 (10)	0.0011 (10)
C20	0.0358 (10)	0.0407 (10)	0.0379 (9)	0.0033 (8)	0.0053 (8)	0.0021 (8)
C21	0.0420 (11)	0.0397 (10)	0.0493 (11)	0.0052 (8)	0.0064 (9)	0.0112 (9)
C22	0.0339 (10)	0.0401 (10)	0.0457 (11)	0.0027 (8)	0.0027 (8)	0.0022 (8)
C23	0.073 (4)	0.058 (4)	0.089 (4)	−0.030 (3)	0.020 (4)	−0.011 (3)
C23'	0.071 (6)	0.042 (4)	0.092 (6)	−0.006 (4)	0.018 (4)	0.004 (4)
C24	0.084 (4)	0.068 (4)	0.090 (4)	−0.025 (3)	0.029 (3)	−0.022 (3)
C24'	0.070 (5)	0.075 (5)	0.091 (6)	−0.024 (4)	0.010 (5)	−0.005 (4)
C25	0.0613 (16)	0.108 (2)	0.0788 (17)	0.0450 (15)	0.0224 (14)	0.0225 (15)
C26	0.0531 (15)	0.0748 (16)	0.104 (2)	0.0225 (12)	0.0098 (14)	0.0167 (14)

### Geometric parameters (Å, °)

**Table:**

O2—C21	1.198 (2)	C14—C15	1.380 (3)
O1—C21	1.321 (2)	C14—C19	1.390 (3)
O1—C23'	1.451 (8)	C15—C16	1.381 (3)
O1—C23	1.486 (6)	C15—H15	0.9300
O4—C22	1.196 (2)	C16—C17	1.372 (3)
O3—C22	1.333 (2)	C16—H16	0.9300
O3—C25	1.459 (3)	C17—C18	1.367 (3)
C1—C2	1.540 (2)	C17—H17	0.9300
C1—C14	1.547 (2)	C18—C19	1.374 (3)
C1—C8	1.554 (2)	C18—H18	0.9300
C1—C20	1.585 (2)	C19—H19	0.9300
C2—C3	1.390 (3)	C20—C22	1.521 (2)
C2—C7	1.393 (2)	C20—C21	1.522 (3)
C3—C4	1.384 (3)	C20—H20	0.9800
C3—H3	0.9300	C23—C24	1.491 (7)
C4—C5	1.373 (3)	C23—H23A	0.9700
C4—H4	0.9300	C23—H23B	0.9700
C5—C6	1.376 (3)	C23'—C24'	1.492 (9)
C5—H5	0.9300	C23'—H23C	0.9700
C6—C7	1.377 (3)	C23'—H23D	0.9700
C6—H6	0.9300	C24—H24A	0.9600
C7—H7	0.9300	C24—H24B	0.9600
C8—C13	1.377 (3)	C24—H24C	0.9600
C8—C9	1.392 (3)	C24'—H24D	0.9600
C9—C10	1.370 (3)	C24'—H24E	0.9600
C9—H9	0.9300	C24'—H24F	0.9600
C10—C11	1.376 (3)	C25—C26	1.456 (3)
C10—H10	0.9300	C25—H25A	0.9700
C11—C12	1.358 (3)	C25—H25B	0.9700
C11—H11	0.9300	C26—H26A	0.9600
C12—C13	1.396 (3)	C26—H26B	0.9600
C12—H12	0.9300	C26—H26C	0.9600
C13—H13	0.9300		
			
C21—O1—C23'	124.0 (6)	C16—C17—H17	120.3
C21—O1—C23	112.0 (3)	C17—C18—C19	120.1 (2)
C22—O3—C25	116.18 (16)	C17—C18—H18	119.9
C2—C1—C14	114.55 (14)	C19—C18—H18	119.9
C2—C1—C8	109.59 (14)	C18—C19—C14	121.6 (2)
C14—C1—C8	104.36 (13)	C18—C19—H19	119.2
C2—C1—C20	108.83 (13)	C14—C19—H19	119.2
C14—C1—C20	108.08 (14)	C22—C20—C21	108.85 (15)
C8—C1—C20	111.40 (14)	C22—C20—C1	115.39 (14)
C3—C2—C7	117.29 (17)	C21—C20—C1	112.30 (14)
C3—C2—C1	121.21 (15)	C22—C20—H20	106.6
C7—C2—C1	121.27 (15)	C21—C20—H20	106.6
C4—C3—C2	121.24 (18)	C1—C20—H20	106.6
C4—C3—H3	119.4	O2—C21—O1	123.98 (19)
C2—C3—H3	119.4	O2—C21—C20	123.97 (18)
C5—C4—C3	120.32 (19)	O1—C21—C20	112.05 (16)
C5—C4—H4	119.8	O4—C22—O3	123.32 (17)
C3—C4—H4	119.8	O4—C22—C20	128.13 (17)
C4—C5—C6	119.38 (19)	O3—C22—C20	108.54 (15)
C4—C5—H5	120.3	O1—C23—C24	108.3 (5)
C6—C5—H5	120.3	O1—C23—H23A	110.0
C5—C6—C7	120.44 (19)	C24—C23—H23A	110.0
C5—C6—H6	119.8	O1—C23—H23B	110.0
C7—C6—H6	119.8	C24—C23—H23B	110.0
C6—C7—C2	121.30 (18)	H23A—C23—H23B	108.4
C6—C7—H7	119.3	O1—C23'—C24'	103.5 (7)
C2—C7—H7	119.3	O1—C23'—H23C	111.1
C13—C8—C9	117.30 (17)	C24'—C23'—H23C	111.1
C13—C8—C1	125.47 (17)	O1—C23'—H23D	111.1
C9—C8—C1	117.22 (16)	C24'—C23'—H23D	111.1
C10—C9—C8	121.7 (2)	H23C—C23'—H23D	109.0
C10—C9—H9	119.1	C23—C24—H24A	109.5
C8—C9—H9	119.1	C23—C24—H24B	109.5
C9—C10—C11	120.4 (2)	H24A—C24—H24B	109.5
C9—C10—H10	119.8	C23—C24—H24C	109.5
C11—C10—H10	119.8	H24A—C24—H24C	109.5
C12—C11—C10	119.0 (2)	H24B—C24—H24C	109.5
C12—C11—H11	120.5	C23'—C24'—H24D	109.5
C10—C11—H11	120.5	C23'—C24'—H24E	109.5
C11—C12—C13	121.1 (2)	H24D—C24'—H24E	109.5
C11—C12—H12	119.5	C23'—C24'—H24F	109.5
C13—C12—H12	119.5	H24D—C24'—H24F	109.5
C8—C13—C12	120.6 (2)	H24E—C24'—H24F	109.5
C8—C13—H13	119.7	C26—C25—O3	108.75 (19)
C12—C13—H13	119.7	C26—C25—H25A	109.9
C15—C14—C19	117.23 (18)	O3—C25—H25A	109.9
C15—C14—C1	124.25 (16)	C26—C25—H25B	109.9
C19—C14—C1	118.43 (16)	O3—C25—H25B	109.9
C14—C15—C16	121.1 (2)	H25A—C25—H25B	108.3
C14—C15—H15	119.5	C25—C26—H26A	109.5
C16—C15—H15	119.5	C25—C26—H26B	109.5
C17—C16—C15	120.5 (2)	H26A—C26—H26B	109.5
C17—C16—H16	119.8	C25—C26—H26C	109.5
C15—C16—H16	119.8	H26A—C26—H26C	109.5
C18—C17—C16	119.4 (2)	H26B—C26—H26C	109.5
C18—C17—H17	120.3		
			
C14—C1—C2—C3	138.03 (17)	C19—C14—C15—C16	−2.7 (3)
C8—C1—C2—C3	21.2 (2)	C1—C14—C15—C16	−179.21 (18)
C20—C1—C2—C3	−100.89 (18)	C14—C15—C16—C17	0.4 (3)
C14—C1—C2—C7	−47.6 (2)	C15—C16—C17—C18	1.5 (4)
C8—C1—C2—C7	−164.48 (15)	C16—C17—C18—C19	−1.0 (4)
C20—C1—C2—C7	73.48 (19)	C17—C18—C19—C14	−1.4 (3)
C7—C2—C3—C4	1.5 (3)	C15—C14—C19—C18	3.2 (3)
C1—C2—C3—C4	176.05 (17)	C1—C14—C19—C18	179.89 (18)
C2—C3—C4—C5	−0.2 (3)	C2—C1—C20—C22	−89.67 (18)
C3—C4—C5—C6	−0.7 (3)	C14—C1—C20—C22	35.3 (2)
C4—C5—C6—C7	0.2 (3)	C8—C1—C20—C22	149.39 (15)
C5—C6—C7—C2	1.2 (3)	C2—C1—C20—C21	35.86 (19)
C3—C2—C7—C6	−2.0 (3)	C14—C1—C20—C21	160.83 (14)
C1—C2—C7—C6	−176.55 (16)	C8—C1—C20—C21	−85.08 (18)
C2—C1—C8—C13	−115.26 (19)	C23'—O1—C21—O2	10.1 (5)
C14—C1—C8—C13	121.62 (19)	C23—O1—C21—O2	−10.6 (6)
C20—C1—C8—C13	5.2 (2)	C23'—O1—C21—C20	−169.3 (5)
C2—C1—C8—C9	65.5 (2)	C23—O1—C21—C20	170.0 (5)
C14—C1—C8—C9	−57.6 (2)	C22—C20—C21—O2	−153.65 (19)
C20—C1—C8—C9	−173.96 (16)	C1—C20—C21—O2	77.3 (2)
C13—C8—C9—C10	0.1 (3)	C22—C20—C21—O1	25.7 (2)
C1—C8—C9—C10	179.37 (17)	C1—C20—C21—O1	−103.29 (17)
C8—C9—C10—C11	−0.9 (3)	C25—O3—C22—O4	1.2 (3)
C9—C10—C11—C12	0.7 (3)	C25—O3—C22—C20	−177.88 (19)
C10—C11—C12—C13	0.3 (3)	C21—C20—C22—O4	−100.3 (2)
C9—C8—C13—C12	0.9 (3)	C1—C20—C22—O4	27.0 (3)
C1—C8—C13—C12	−178.32 (18)	C21—C20—C22—O3	78.69 (19)
C11—C12—C13—C8	−1.1 (3)	C1—C20—C22—O3	−154.02 (15)
C2—C1—C14—C15	−13.0 (2)	C21—O1—C23—C24	−157.0 (8)
C8—C1—C14—C15	106.85 (19)	C23'—O1—C23—C24	75.1 (19)
C20—C1—C14—C15	−134.48 (18)	C21—O1—C23'—C24'	−103.9 (12)
C2—C1—C14—C19	170.57 (16)	C23—O1—C23'—C24'	−41.9 (15)
C8—C1—C14—C19	−69.6 (2)	C22—O3—C25—C26	−178.7 (2)
C20—C1—C14—C19	49.1 (2)		
